# Cost benefits of rapid recanalization using intraarterial thrombectomy

**DOI:** 10.1002/brb3.830

**Published:** 2017-09-11

**Authors:** Hye Seon Jeong, Jong Wook Shin, Hyon‐Jo Kwon, Hyeon‐Song Koh, Hae‐Sung Nam, Hee Seon Yu, Na Young Yoon, Jei Kim

**Affiliations:** ^1^ Daejeon‐Chungnam Regional Cerebrovascular Center Hospital and School of Medicine Chungnam National University Daejeon Korea; ^2^ Department of Neurology Hospital and School of Medicine Chungnam National University Daejeon Korea; ^3^ Department of Neurosurgery Hospital and School of Medicine Chungnam National University Daejeon Korea; ^4^ Department of Preventive Medicine School of Medicine Chungnam National University Daejeon Korea

**Keywords:** cost‐utility analysis, intraarterial thrombectomy, onset‐to‐recanalization time, quality adjusted‐life year

## Abstract

**Objectives:**

Thrombolytic therapy is associated with favorable clinical outcomes after successful and rapid recanalization in patients with acute ischemic stroke. This study aimed to evaluate the cost benefits and clinical outcomes at 1 year after intraarterial thrombectomy (IAT) by the rapidity of the successful recanalization.

**Materials & Methods:**

Clinical outcomes of and medical costs incurred by 230 patients with acute ischemic stroke who underwent IAT were compared by the rapidity from symptom onset to successful recanalization (2b/3 thrombolysis in cerebral infarction grade): ≤6‐hr (*n* = 143), >6‐hr (*n* = 31), and no‐recanalization (*n* = 56). Clinical outcomes including functional independence (0–2 modified Rankin Score), mortality, and home‐discharge checked at 1 year post‐IAT were compared among the three groups. Cost utility was calculated using quality‐adjusted life years (QALY) estimated using the EuroQol‐5 dimensions‐3 levels questionnaire and the fees paid for institutional rehabilitation during the year post‐IAT, and, was compared among the groups.

**Results:**

Patients in the ≤6‐hr group showed higher functional independence (≤6‐hr, 70%; >6‐hr, 40%; no‐recanalization, 6%, *p *<* *.001) and home‐discharge rate (73%, 52%, 21%, and respectively, *p *<* *.001), and lower mortality (10%, 16%, and 43%, respectively, *p *<* *.001) at 1 year after IAT than other two groups. The cost utility of the ≤6‐hr group was $35,557/QALY higher than that of the >6‐hr group, and $27.829/QALY higher than no‐recanalization group.

**Conclusions:**

Rapid and successful recanalization of the occluded intracranial vessels within 6 hr after the onset of symptoms resulted in markedly higher cost utility and functional independence at 1 year post‐IAT.

## 1 INTRODUCTION

Thrombolytic therapy is associated with favorable clinical outcomes after successful recanalization in patients with acute ischemic stroke (Rha & Saver, 2007). Intraarterial thrombectomy (IAT) clinically improves patients with acute ischemic stroke by allowing rapid recanalization of occluded intracranial vessels (Berkhemer et al., 2015; Campbell et al., 2015; Goyal et al., 2015; Jovin et al., 2015; Saver et al., 2015). Despite its clinical success, the direct cost‐effectiveness of IAT remains unexplored.

While IAT is more expensive and requires specialized personnel, its high cost is justified by the improved lifetime clinical outcomes and cost‐effectiveness (Aronsson, Persson, Blomstrand, Wester, & Levin, 2016; Ganesalingam et al., 2015; Leppert, Campbell, Simpson, & Burke, 2015). However, the long‐term (>3 months post‐IAT) clinical and cost benefits for patients stratified according to the time from symptom onset to recanalization remain unclear.

The aim of this study was to evaluate costs and clinical outcomes at discharge after acute stroke management and 1‐year post‐IAT. The costs and changes in clinical outcomes were compared based on the symptom onset‐to‐recanalization of the occluded vessels.

## 2 MATERIALS AND METHODS

### 2.1 Patients

We reviewed the clinical records and medical costs of 230 patients with acute ischemic stroke who underwent IAT to recanalize occluded intracranial vessels, from October 2010 to May 2015. The inclusion criteria were: (1) age >18 years, (2) arrival at emergency room (ER) within 6 hr of symptom onset, (3) baseline National Institutes of Health Stroke Scale (NIHSS) score ≥4 (Brott et al., 1989), and (4) unilateral middle cerebral artery (MCA) and/or terminal internal carotid artery occlusion confirmed by cerebral angiography. The study protocol was approved by the Institutional Review Board of a University Hospital and conformed to the tenets of the Declaration of Helsinki; the requirement for informed consent was waived because of the retrospective nature of the study.

### 2.2 Critical pathways for intraarterial thrombectomy

IAT was performed as a bridging therapy after intravenous thrombolysis (IVT) or as primary therapy, following the critical pathway established at the Regional Cerebrovascular Center of a University Hospital (Jeong et al., 2015). Briefly, patients who arrived at the ER within 4.5 hr of symptom onset without hemorrhage or significant low density on the initial computed tomography (CT) scan (i.e., less than one‐third of the MCA territory) received intravenous recombinant tissue plasminogen activator (does, 0.9 mg/kg). If the patient exhibited no clinical improvement after IVT, and their diffusion‐ and perfusion‐weighted images on magnetic resonance imaging (MRI) mismatched, IAT was performed as bridging therapy. Patients who arrived within 6 hr of symptom onset, but were not IVT candidates, were treated with IAT after confirming the absence of hemorrhage on the initial CT scan and the presence of diffusion‐/perfusion‐weighted image mismatch on MRI. For patients having unclear symptom onset including wake‐up stroke, last normal time (LNT) was considered as the symptom onset time. If an unclear‐onset patient arrived at ER within 4.5 hr of LNT, IVT was considered for the patient. If an unclear‐onset‐patient arrived within 6 hr from LNT and showed marked perfusion‐diffusion mismatch on initial MRI, IAT was considered for the patient. The thrombus was removed during IAT using a retrievable stent (Solitaire AB, ev3; Covidien, Dublin, Ireland) or a suction device (Penumbra System; Penumbra Inc., Alameda, CA, USA) (Jeong et al., 2015). Balloon angioplasty and/or stenting were performed if recanalized arteries became re‐occluded post‐IAT.

### 2.3 Clinical and functional outcome evaluations after IAT

Patients’ demographic characteristics and cardiovascular risk factors were reviewed. Stroke severity and clinical improvement were assessed based on NIHSS score immediately after ER arrival, and immediately, 24‐hr, and 3‐days post‐IAT, and at discharge after acute stroke management. The symptom onset‐to‐recanalization time was calculated as the time from symptom onset to successful recanalization (defined as thrombolysis in cerebral infarction grade 2b/3) (Roth et al., 2010) post‐IAT (Higashida et al., 2003). Symptomatic intracerebral hemorrhage (ICH) was defined as a large hematoma associated with a ≥ 4‐point increase in NIHSS score within 24 hr post‐IAT (Khatri, Wechsler, & Broderick, 2007).

Functional outcomes were assessed according to the modified Rankin Scale (mRS) (Banks & Marotta, 2007) at discharge, 3‐months, and 1‐year post‐IAT. mRS scores of 0–2 were considered a good clinical outcome with functional independence. Hospitalization for acute stroke care, including IAT, and for institutional rehabilitation for 1 year post‐IAT was evaluated. Discharge placement (home, rehabilitation institute, nursing facility providing general care, and death) was evaluated after acute stroke care, including IAT, and 1‐year post‐IAT.

### 2.4 Costs

Medical costs were divided into (1) direct costs of acute stroke care, including IAT, and (2) costs of rehabilitation care for up to 1 year after acute management. Both acute and rehabilitation care costs were paid based on payment recommendations developed by the National Health Insurance Service (NHIS) of South Korea. The NHIS acute stroke care cost was developed based on the fee‐for‐service (FFS) system in acute care hospitals. Rehabilitation care costs were based on the co‐payment system combined with a diagnosis‐related group (DRG) and case‐payment.

Individual patient's acute management costs were obtained by reviewing their billing records at discharge. Acute care costs included fees for IAT and acute stroke care, including those for the stroke unit and/or ward, neurological/physical examination, bed‐side rehabilitation therapy, medications and injections, hemicraniectomy, laboratory tests, and imaging studies, including roentgenogram, CT, MRI, transcranial Doppler, and carotid duplex sonography. Rehabilitation care costs were obtained using the daily co‐payment rehabilitation costs and total duration of stay in the rehabilitation institute. Rehabilitation care costs were obtained only for survivors who received rehabilitation therapy during the 1 year post‐IAT (the maximum admission period in the rehabilitation institute).

### 2.5 Cost‐utility analysis

To determine the cost utility, stratified by the onset‐to‐recanalization interval, the quality of life of survivors at 1‐year post‐IAT was first assessed using the quality‐adjusted life years (QALYs), which indicates mortality and differences in the health‐related quality of life (Rabin & de Charro, 2001) of patients based on the Korean version of the EuroQol‐5 Dimensions‐3 Level (EQ‐5D‐3L) questionnaire (Kim, Cho, Uhm, Kim, & Bae, 2005). Responses to the EQ‐5D‐3L were obtained from patients or their family members (when the patients could not communicate) by phone at 1‐year post‐IAT. The individual utility weights for the EQ‐5D‐3L‐based QALY were obtained from a South Korean general population survey (Lee et al., 2009). The direct saving of the institutional rehabilitation costs and the QALY gains were obtained for the >6‐hr and ≤6‐hr groups, by comparison with the 1‐year cost and QALY‐gain of the no‐recanalization group. The cost‐utility ratio (CUR) of patients in the ≤6‐hr and >6‐hr groups was calculated using the formula: $\begin{array}{c} (\text{CUR}=\left[{\text{Cost}}_{\leq 6-\mathrm{hrs}-\mathrm{or}>6-\mathrm{hrs}-\mathrm{recanalization}}-{\text{Cost}}_{\mathrm{no}-\mathrm{recanalization}}\right]/ \end{array}$ $\begin{array}{c} \left[{\text{QALY}}_{\leq 6-\mathrm{hrs}-\mathrm{or}>6-\mathrm{hrs}-\mathrm{recanalization}}-{\text{QALY}}_{\mathrm{no}-\mathrm{recanalization}}]\right) \end{array}$ (Cohen & Reynolds, 2008).

### 2.6 Statistical analysis

Differences in clinical and radiological variables, including age, sex, risk factors, initial NIHSS score, onset‐to‐arrival time, IVT before IAT, stroke subtypes, and occlusion site, were analyzed using analysis of variance (ANOVA) with Scheffe's post‐hoc tests or chi‐square test. Differences in the length of stay, QALY, and mean rehabilitation care costs at post‐acute care discharge and at 1‐year post‐institutional rehabilitation were also compared among the three groups by ANOVA. Differences in the rates of complication development, placement after acute management and 1‐year post‐IAT, and functional independence were compared among the three groups using ANOVA or chi‐square test. Multiple logistic regression analysis was performed using all clinical and radiological variables from the univariate analyses to determine independent factors related to home discharge after 1 year. All statistical analyses were performed using SPSS version 22.0 (IBM SPSS Statistics, Chicago, IL, USA). Statistical significance was set at *p *≤* *.05.

## 3 RESULTS

### 3.1 Baseline characteristics

The clinical outcome and medical cost data of 230 consecutive patients who underwent IAT (141 males; mean age ± standard deviation, 68.6 ± 12.4 years) were compared among the ≤6‐hr recanalization (*n* = 143), >6‐hr recanalization (*n* = 31), and no‐recanalization groups (*n* = 56) (Table 1). Age of onset was lower in the ≤6‐hr recanalization group (*p *=* *.006), but the proportion of male subjects was similar among the three groups (*p *=* *.559). The history of previous stroke was less in the ≤6‐hr recanalization (*p *=* *.032), but the incidence of hypertension, diabetes, and atrial fibrillation was not significantly different (*p *=* *.159, *p *=* *.293, and *p *=* *.660, respectively). The onset‐to‐arrival time was 1.5 hr in the ≤6‐hr recanalization group, 2 hr in the no‐recanalization group, and about 5 hr in the >6‐hr recanalization group (*p *<* *.001). Thus, IVT was performed pre‐IAT in half of the patients in the ≤6‐hr recanalization (49%) and no‐recanalization (43%) groups, and in only 16% of the patients in the >6‐hr recanalization group (*p *=* *.003). The initial NIHSS scores and the distribution of stroke subtypes did not differ among the 3 groups. Initial MR angiography revealed that MCA occlusion was more frequent in the ≤6‐ hr recanalization group than in the other two groups (*p *=* *.008).

**Table 1:** Baseline characteristics

	Onset‐to‐recanalization		No‐recanalization (*n* = 56, %)	*p*‐value
	≤6‐hr (*n* = 143, %)	>6‐hr (*n* = 31, %)		
Age (mean ± *SD*)	66.8 ± 13.2	69.1 ± 10.7	73.0 ± 10.1	.006
Sex, male:female (%)	91:52 (64:36)	19:12 (61:39)	31:25 (55:45)	.559
Diabetes mellitus	24 (17)	8 (26)	14 (25)	.293
Hypertension	73 (51)	17 (55)	37 (66)	.159
Atrial fibrillation	82 (57)	15 (49)	31 (55)	.660
Previous stroke	17 (12)	8 (26)	14 (25)	.032
Initial NIHSS score	11.5 ± 4.4	11.5 ± 3.2	12.2 ± 4.3	.546
Onset‐to‐arrival time	98.1 ± 62.8	271.5 ± 115.6	130.0 ± 103.7	<.001
Intravenous thrombolysis	70 (49)	5 (16)	24 (43)	.003
Stroke classification				
Large artery atherosclerosis	41 (29)	9 (29)	21 (38)	.754
Cardioembolism	84 (59)	18 (58)	31 (55)	
Undetermined	13 (9)	2 (7)	2 (4)	
Other determined	5 (4)	2 (7)	2 (4)	
Occluded artery				
Terminal‐intracranial artery	25 (18)	9 (29)	23 (41)	.008
Middle cerebral artery	97 (68)	16 (52)	25 (45)	
Basilar artery	21 (15)	6 (19)	8 (14)	
Intracranial angioplasty/stenting				
No	134 (94)	25 (81)	43 (77)	<.001
Angioplasty only	2 (1)	1 (3)	10 (18)	
Angioplasty + stenting	7 (5)	5 (16)	3 (5)	

*SD*, standard deviation; NIHSS, National Institute of Health Stroke Scale.

### 3.2 Clinical outcome differences

Hospital stay for acute stroke care was significantly shorter (9.8 ± 5.8 days) in the ≤6‐ hr recanalization group than in the other two groups (>6‐hr, 13.2 ± 8.1 days; no‐recanalization, 16.7 ± 19.3 days, *p *=* *.001, Table 2). The development of symptomatic ICH, aspiration pneumonia, or other medical complications did not differ significantly among the groups (Table 2). Clinical outcome analysis revealed that functional independence (0–2 mRS) at discharge (≤6‐hr, 57%; >6‐hr, 23%; no recanalization, 0) (*p *<* *.001), after 3 months (≤6‐hr, 71%; >6‐hr, 42%; no recanalization, 2%) (*p < *.001), and after 1 year (≤6‐hr, 70%; >6‐hr, 40%; no recanalization, 6%) (*p *<* *.001) was significantly higher in the ≤6‐hr recanalization than in the other groups. However, the mortality rates at discharge (≤6‐hr, 4%; >6‐hr, 3%; no recanalization, 29%) and after 1 year (≤6‐hr, 10%; >6‐hr, 16%; no recanalization, 43%) were significantly higher (*p *<* *.001) in the no‐recanalization group than in the other groups. Forty‐eight percent of the patients in the rapid recanalization group (≤6‐hr) went home after discharge, while 16% and 0% of the patients in the late‐ (>6‐hr) and no‐recanalization groups went home after discharge (*p *<* *.001). One year post‐IAT, 72% of the patients in the rapid recanalization group (≤6‐hr) were staying at home, compared to 50% and 21% of the patients in the late‐ (>6‐hr) and no‐recanalization groups (*p *<* *.001).

**Table 2:** Clinical outcome differences according to the onset‐to‐recanalization time after intraarterial thrombectomy (IAT)

	Onset‐to‐recanalization		No‐recanalization (*n* = 56, %)	*p*‐value
	≤6‐hr (*n* = 143, %)	>6‐hr (*n* = 31, %)		
Length of stay before discharge (days ± *SD*)	9.8 ± 5.8	13.2 ± 8.1	16.7 ± 19.3	.001
Complication in acute period				
Symptomatic intracerebral hemorrhage	3 (2)	2 (6)	3 (5)	.330
Aspiration pneumonia	16 (11)	3 (10)	8 (14)	.496
Other medical complications^a^	8 (6)	3 (10)	2 (4)	.549
Placement after acute management				
Home	68 (48)	5 (16)	0	<.001
Rehabilitation institute	53 (37)	19 (61)	24 (43)	
Nursing facility	16 (11)	6 (19)	16 (29)	
Expired	6 (4)	1 (3)	16 (29)	
Placement at 1 year after IAT				
At home	104 (73)	16 (52)	12 (21)	<.001
Rehabilitation or nursing facility	23 (16)	9 (29)	20 (36)	
Expired	14 (10)	5 (16)	24 (43)	
Follow‐up loss	2 (1)	1 (3)	0	
Functional independence (modified Rankin scale 0–2)				
At discharge	81 (57)	7 (23)	0	<.001
3‐month	102 (71)	13 (42)	1 (2)	<.001
1‐year	99 (70)	12 (40)	3 (6)	<.001

*SD*, standard deviation.

a Other medical complications include stress induced cardiomyopathy, ischemic heart disease, urinary tract infection, and sepsis.

Multiple regression analysis revealed that a younger age (*p *=* *.009), Male sex (*p *=* *.029), low initial NIHSS score (*p *=* *.030), and no medical complications (*p *=* *.002) could independently predict home‐staying at 1‐year post‐IAT (Table 3). Recanalization was associated with higher odds (≤6‐hr, odds ratio 8.3, *p *<* *.001; >6‐hr, odds ratio 2.5, *p *=* *.047) for home‐staying at 1‐year post‐IAT, than for no recanalization (Table 3).

**Table 3:** Factors predicting discharge within 1 year of intraarterial thrombectomy (IAT)

	Odds ratio	95% Confidential interval		*p*‐value
		Lower limit	Upper limit	
Age	0.955	0.923	0.988	.009
Male sex	2.187	1.082	4.420	.029
Hypertension	1.093	0.518	2.309	.815
Diabetes mellitus	1.168	0.475	2.869	.736
Atrial fibrillation	1.051	0.516	2.141	.890
Previous stroke	1.226	0.489	3.074	.664
Initial NIHSS	0.904	0.825	0.990	.030
MCA occlusion	1.224	0.583	2.567	.593
Medical complication	0.241	0.099	0.586	.002
Severe hemorrhage after IAT	0.548	0.140	2.142	.387
Recanalization after IAT				
Unsuccessful recanalization	Reference			
Recanalization >6‐hr	3.052	1.015	9.170	.047
Recanalization ≤6‐hr	8.055	3.360	19.312	<.001

NIHSS, National Institute of Health Stroke Scale; MCA, middle cerebral artery.

### 3.3 Cost utility

The overall cost for IAT intervention and acute stroke management for the ≤6‐hr recanalization group (mean, $9,191) was lower (*p *=* *.004) than that for the >6‐hr recanalization (mean, $12,159) and no‐recanalization (mean, $11,918) groups (Table 4). The costs for IAT intervention was higher in >6‐hr and no recanalization than ≤6‐hr groups (*p *=* *.092). However, hospital room (*p *=* *.006), medication and injections (*p *=* *.012), laboratory tests (*p *<* *.001), bed‐side rehabilitation (*p *=* *.012), and hemicraniectomy (*p *=* *.016) were significantly lower in the ≤6‐hr recanalization group than in the other groups (Table 4).

**Table 4:** Differences in costs paid for intraarterial thrombectomy (IAT) and acute stroke management according to recanalization time

Cost items ($)	Onset‐to‐recanalization		No‐recanalization (*n* = 56)	*p*‐value
	≤6‐hr (*n* = 143)	>6‐hr (*n* = 31)		
Room	631 ± 583	1,231 ± 1,066	1,107 ± 1,504	.006
Neurological/Physical examination	71 ± 21	66 ± 14	68 ± 13	.597
Medication/injection	72 ± 13	238 ± 449	141 ± 267	.012
Bed‐side rehabilitation	60 ± 82	89 ± 100	108 ± 109	.012
Operation‐related^a^	2,383 ± 1,181	3,497 ± 3,194	3,364 ± 2,982	.016
IAT‐related^b^	3,484 ± 116	3,802 ± 1,483	4,135 ± 2,046	.094
Laboratory test^c^	903 ± 598	1,614 ± 1,139	1,540 ± 1,517	<.001
Imaging studies^d^	1,497 ± 296	1,637 ± 366	1,453 ± 416	.182
Total	9,101 ± 2,974	12,159 ± 6,884	11,918 ± 7,468	.004

Costs paid in Korean Won (₩) were converted to United States Dollars ($) at the conversion rate of ₩1,150 to the $.

a Operation‐related costs include operation costs for hemicraniectomy to decompress brain edema that developed after IAT.

b IAT‐related costs include costs for intraarterial thrombectomy to recanalize occluded intracranial vessels.

c Laboratory tests include blood tests (for complete blood count, chemistry, electrolytes, lipid profile, coagulation), urine analysis, and electrocardiography.

d Imaging studies include roentgenogram, brain computed tomography, brain magnetic resonance imaging and magnetic resonance angiography, transcranial Doppler, carotid duplex ultrasonography performed during IAT, and acute stroke management.

Cost utility for 1 year after IAT was analyzed for 184 survivors (≤6‐hr, 127 patients; >6‐hr, 25 patients; no recanalization, 32 patients; Table 5) at 1 year post‐IAT. The length of stay at a rehabilitation institute 1‐year post‐IAT differed significantly (*p *<* *.001) among groups (≤6‐hr, 3 months; >6‐hr, 4.5 months; no recanalization, 7.5 months; Table 5). The QALY gain (compared to the no‐recanalization group) at 1‐year post‐IAT was 0.48 for the ≤6‐hr group and 0.34 for the >6‐hr group. The overall fees directly saved (in comparison with the no‐recanalization group [$29,382]) for the rehabilitation stay at 1‐year post‐IAT was 45% in the ≤6‐hr group ($16,024) and 29% in the >6‐hr group ($21,022). Thus, patients in the ≤6‐hr group saved $27.829/QALY as compared to the no‐recanalization group, while those from the >6‐hr group saved $24,647/QALY (Table 5). Patients in the ≤6‐hr group gained 0.14 QALY and directly saved $4,978 more than patients in the >6‐hr group; the cost utility of the ≤6‐hr group was $35,557/QALY higher than that of the >6‐hr group.

**Table 5:** Cost effectiveness analysis of intraarterial thrombectomy (IAT) among the 1‐year survivors according to the onset‐to‐recanalization time

	Onset‐to‐recanalization		No‐recanalization (*n* = 32)	*p*‐value
	≤6‐hr (*n* = 127)	>6‐hr (*n* = 25)		
Length of stay (days) in the rehabilitation institute at 1 year after IAT (mean ± *SD*)	87.2 ± 136.4	133.7 ± 152.4	224.3 ± 130.3	<.001
Costs (in $) for institutional rehabilitation 1 year after discharge (mean ± *SD*)	$16,024 ± 12,320	$21,002 ± 15,504	$29,382 ± 17,403	<.001
Direct institutional rehabilitation cost saving (compared to the no‐recanalization group)^b^	−$13,357	−$8,380	Baseline	
QALY at 1 year after discharge (mean ± *SD*)	0.80 ± 0.34	0.66 ± 0.39	0.32 ± 0.43	<.001
QALY gained (compared to the no‐recanalization group)^a^	0.48	0.34	Baseline	
Cost‐utility ratio (compared to the no‐recanalization group) (=a/b)	−$27,829/QALY	−$24,647/QALY	Baseline	
Direct institutional rehabilitation cost saving (compared to the >6‐hr group)^b^	−4,978	Baseline	NA	
QALY gained (compared to the >6‐hr group)^b^	0.14	Baseline	NA	
Cost‐utility ratio (compared to the >6‐hr group) (=c/d)	−$35,557	Baseline	NA	

QUALY, quality‐adjusted life years.

Costs paid in Korean Won (₩) were converted to United States Dollars ($) at the conversion rate of ₩1,150 to the $.

Direct cost saving of Recanalization groups = (mean rehabilitation costs of ≤6‐hr or >6‐hr Recanalization groups)−(mean rehabilitation costs of No‐recanalization group).

QALY gained of Recanalization groups = (QALY of ≤6‐hr or >6‐hr Recanalization groups)−(QALY of No‐recanalization group).

a Direct cost saving of ≤6‐hr Recanalization group = (mean rehabilitation costs of ≤6‐hr Recanalization groups)−(mean rehabilitation costs of or >6‐hr Recanalization group).

b QALY gained ≤6‐hr Recanalization group = (QALY of ≤6‐hr Recanalization groups)−(QALY of or >6‐hr Recanalization group).

## 4 DISCUSSION

We determined the long‐term cost and functional benefits at 1‐year post‐IAT in patients with acute ischemic stroke. Previous studies have described short‐term (measured at 3 months) clinical benefits post‐IAT (Berkhemer et al., 2015; Campbell et al., 2015; Goyal et al., 2015; Jovin et al., 2015; Saver et al., 2015), and statistically estimated the lifelong cost‐benefits of IAT (Aronsson et al., 2016). We evaluated the long‐term functional outcome and cost utility at 1‐year post‐IAT by stratifying the study sample based on the symptom onset‐to‐recanalization interval. Patients recanalized ≤6‐hr after symptom onset improved significantly in functional independence and home‐staying at 1‐year post‐IAT. The cost utility paid for institutional rehabilitation at 1‐year post‐IAT was significantly different between patients with or without successful recanalization.

Our study used 6 hr as the threshold time for recanalization for classifying patients undergoing IAT. Shorter onset‐to‐recanalization intervals in patients with acute ischemic stroke are associated with better clinical outcomes (Jeong et al., 2015). Previous reports have used onset‐to‐recanalization time windows of ≤6‐hr (Berkhemer et al., 2015) or ≤8‐hr (Jovin et al., 2015) to evaluate the functional outcomes of IAT. For example, Jeong et al. recommended 6 hr as the threshold for the onset‐to‐recanalization interval in order to achieve a 50% chance of functional independence at 3 months after IAT (Jeong et al., 2015).The optimal time‐window for achieving favorable clinical outcomes remains debated; we chose to use 6 hr.

Jørgensen et al. suggested that reliable functional recovery can be achieved within 12 weeks from the onset of stroke‐related symptoms (Jørgensen et al., 1995a). However, we found that the functional independence measured at 3 months remained unchanged at 1 year post‐IAT. Therefore, we chose a 1‐year follow‐up period to evaluate reliable functional outcomes post‐IAT. While the home‐discharge rate at 1‐year post‐IAT was 73% in the ≤6‐hr recanalization group, it was only 52% and 21% in the >6‐hr and no‐recanalization groups. In a previous study that evaluated discharge placement as a function of the initial stroke severity, 14% of the patients with very severe stroke (defined as Scandinavian Stroke Scale [SSS], 0‐14; NIHSS score, 25–18 converted using a fitted model (Ali, Cheek, Sills, Crome, & Roffe, 2007) were discharged home, whereas 34%, 74%, and 93% of the patients with severe (SSS, 15–29; NIHSS score, 1.5–10.5), moderate (SSS, 30–44; NIHSS score, 10–3), and mild (SSS, 45–58; NIHSS score, 2.5–0) strokes were discharged home (Jørgensen et al., 1995b). The majority of our patients presented with severe strokes (mean NIHSS score, 11.5–12.2). However, after IAT, the home‐discharge rate of patients in the ≤6‐hr group increased to that of patients with moderately severe strokes. On the other hand, the home‐discharge rates of patients in the >6‐hr and no‐recanalization groups remained similar to those of patients with severe or very severe strokes.

The cost effectiveness of IAT is usually estimated based on the cost for acute management, and then, the long term costs are calculated using the estimated costs for index stroke previously reported in the individual country (Aronsson et al., 2016; Ganesalingam et al., 2015; Leppert et al., 2015). The acute management costs for IAT usually increases the standard costs required for IVT, given the expensive devices used and expert personnel required to perform the IAT (Ganesalingam et al., 2015; Leppert et al., 2015). However, these high costs may be considered acceptable given the incremental lifetime cost‐effectiveness ratio of IAT costs remained within a willingness to pay threshold of ≤$50,000/QALY (Ganesalingam et al., 2015; Leppert et al., 2015). We here separately compared the costs of acute IAT management and those of long‐term institutional rehabilitation after IAT. The acute IAT management costs included costs paid for acute stroke care after IAT, as well as for the IAT intervention itself. The IAT intervention cost was less in the ≤6‐hr recanalization group than in the >6‐hr or no‐recanalization groups. After IAT, the admission period was subsequently shorter in the ≤6‐hr recanalization group (10 days) than in the >6‐hr (13 days) and no‐recanalization (17 days) groups. The costs for hemicraniectomy, hospital room, clinical management, and laboratory work‐up was higher in the >6‐hr and no‐recanalization groups than in the ≤6‐hr recanalization group. Ultimately, the total acute management costs were 30% lower in the ≤6‐hr recanalization group ($9,101) than in the >6‐hr ($12,159) or no‐recanalization ($11,918) groups.

Furthermore, we analyzed long‐term cost utility using QALY (measured using the EQ‐5D‐3L responses) and the cost of institutional rehabilitation of functionally dependent patients during the 1 year after IAT. Patients in the ≤6‐hr group gained 0.14 QALY and directly saved $4,978 more than did patients in the >6‐hr group; the cost utility of the ≤6‐hr group was $35,557/QALY higher than that of the >6‐hr group. We found that recanalization of the occluded vessels in <6‐hr results in significant cost utility by improving functional independence and shortening the institutional rehabilitation period post‐IAT.

The present study revealed the importance of rapid recanalization in <6‐hr in reducing the economic burden caused by long‐term institutional rehabilitation after IAT. To increase the likelihood of rapid recanalization by IAT in patients with acute ischemic stroke, comprehensive stroke centers with experienced personnel should be established, where round‐the‐clock IAT is available. Even though the US and several European countries have established such centers (Gorelick, 2013; Leys, Ringelstein, Kaste, & Hacke, 2007), other countries face economic and expertise‐related limitations. Governments, including that of South Korea, should rapidly establish comprehensive stroke centers (Kim et al., 2014).

The present long‐term cost analysis has several limitations due to its retrospective study design. South Korea has been using the co‐payment system combined with DRG and case‐payment for long‐term institutional rehabilitation costs. Thus, retrospective cost analysis cannot evaluate costs incurred towards individual services, such as rehabilitation, medication, injection, laboratory tests, and imaging tests. We did not include indirect costs, such as loss of income and social benefit payments (Saka, McGuire, & Wolfe, 2009). Moreover, the costs of evaluating and treating recurrent stroke in the included patients were not included in the cost analysis. Thus, the overall socioeconomic benefits of IAT could not be analyzed in the present study. Although we describe the actual costs incurred towards acute and long‐term management of IAT, rather than estimating the cost effectiveness of IAT, further studies, including individual service costs, indirect costs, recurrence‐related costs, and direct long‐term costs, are needed to evaluate the overall socioeconomic benefits of IAT.

## 5 CONCLUSIONS

The present study demonstrated the long‐term functional and economic benefits of IAT at 1 year in patients with acute ischemic stroke stratified according to the onset‐to‐recanalization time. Rapid recanalization (≤6‐hr) could affect functional independence in 70% of the patients, which is significantly more than for the >6‐hr and no‐recanalization groups. Therefore, the admission duration for acute management and the chance and duration of institutional rehabilitation was significantly reduced in the ≤6‐hr recanalization group. Finally, the cost utility of the ≤6‐hr recanalization group was higher than that of the >6‐hr recanalization group.

## DISCLOSURES

None.

## CONFLICT OF INTEREST

The authors report no conflict of interest.
